# Prognostic Role of C-Reactive Protein In Urological Cancers: A Meta-Analysis

**DOI:** 10.1038/srep12733

**Published:** 2015-08-03

**Authors:** Liang Zhou, Xiang Cai, Qiang Liu, Zhong-Yu Jian, Hong Li, Kun-Jie Wang

**Affiliations:** 1 Department of Urology, West China Hospital, Sichuan University, Chengdu, Sichuan, China

## Abstract

Growing evidence suggests serum C-reactive protein (CRP) can serve as a prognostic marker in urological cancers. However, some studies yield contradictory results. Our objective was to determine the relationship between baseline serum CRP and survival outcome in urological cancers. We searched PubMed and EMBASE databases until October 2014 without language restrictions. 44 independent studies investigating the association between baseline serum CRP and cancer-specific survival (CSS) or overall survival (OS) were selected. High CRP yielded a worse survival in renal cell carcinoma, prostate cancer, bladder cancer, and upper urinary tract urothelial carcinoma. Combined results of meta-analyses indicated that CRP was a prognostic factor in urological cancers (CSS: p < 0.01; OS: p < 0.01). Subgroup analyses confirmed the significant association between CRP and prognosis, regardless of race and cutoff value of CRP. Specifically, prognostic impact of CRP was also noted in patients with localized RCC treated with nephrectomy (CSS: p < 0.01) and metastatic RCC treated with molecular-targeted therapy (OS: p < 0.01). In conclusion, serum CRP is an independent prognostic factor in urological cancers and risk stratification by serum CRP level could be helpful for prognostic assessment.

Urological cancer comprises of the highest prevalence groups in the world and represents a growing burden on human healthcare^1^,^2^. Renal cell carcinoma (RCC) accounts for approximately 2–3% of all adult malignancies^1^,^3^. Prostate cancer is one of the most common cancers in men and the fifth most common cancer overall^4^. In bladder cancer, which ranks as the ninth most frequent malignancy, an estimated 386,300 new cases and 150,200 deaths from bladder cancer occurred in 2008 worldwide^1^. Despite the great progress in treatment of urological cancers, such as chemotherapy and molecular therapy, the clinical outcome remains not promising due to low objective response rate, tumor local recurrence or distal metastasis. In this condition, identification of patients who are prone to have a poor prognosis has become increasingly important for treatment selection and prognostic estimation. TNM classification and histological grade are clinically most-used prognostic indicators in malignancies. However, the accuracy of TNM classification remains poor in cancers and pathologic analysis for prognosis would miss any information associated patient-related factors^5^. This leaves a considerable space for the development of supplementary biomarkers to predict the prognosis of patients.

It is well documented that inflammation is a hallmark feature in the development and progression of cancer^6^. Increasing evidence suggests that inflammation can help incipient neoplasias to acquire hallmark capabilities and promote tumour growth and metastatic dissemination by sustaining a microenvironment^7^,^8^. C-reactive protein is a representative acute-phase protein and serves as a definitive marker for systemic inflammation^9^. In fact, a remarkable association between CRP and the poor survival in malignancies has been demonstrated, such as colorectal and lung cancers^6^,^8^.

During the last decade, numerous studies explored the prognostic impact of CRP in urological cancers, including renal cell cancer, prostate cancer, bladder cancer and upper urinary urothelial carcinoma. Saito and Kihara concluded that CRP is a useful biomarker for urological cancers^10^. However, some studies failed to draw similar conclusions. Recently, several meta-analyses focused on the association between CRP and the survival of renal cell carcinoma or prostate cancer^11^,^12^,^13^, but urothelial cancer and bladder cancer were not taken into account. More importantly, those reports included a relatively small number of studies. Many researchers have done a lot of studies on the prognostic role of CRP in urological cancers so that a growing number of related studies were published. Therefore, to confirm the prognostic significance of baseline serum CRP in urologic cancers, we gathered the available clinical research evidence and carried out a comprehensive meta-analysis. In this study, we applied a stricter inclusion/exclusion criteria, included much more recent studies, and discussed other important factors, especially the cutoff of CRP level.

## Results

### Study selection

**Figure 1:**
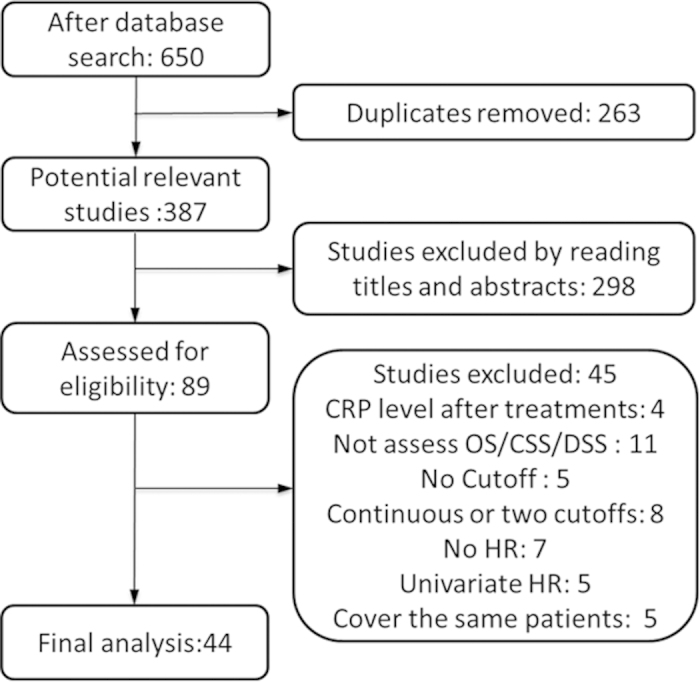
Flow chart of study selection for the meta-analysis.

Figure 1 shows the process of study selection. We identified 650 possibly eligible articles through searching the Pubmed (n = 229) and EMBASE databases (n = 421) but 263 articles were excluded due to duplication. After carefully reviewing the titles and abstracts of 387 articles by the two authors (L.Z, X.C), 89 articles were retained for further examination. Based on the detailed information in full-texts, 45 articles were excluded as documented in Figure 1. Finally, a total of 44 studies comprised 9174 cases were included for our quantitatively analysis.

### Characteristics of included studies

Characteristics of included studies were summarized in Table 1. 44 observational studies were published from 1997 to 2014. All studies met all six points for quality evaluation. Most of these studies were conducted in Asia (n = 31), Europe (n = 11) and North America (n = 2). Of these studies, 6 explored the CRP in the prognosis of prostate cancer^14^,^15^,^16^,^17^,^18^,^19^, 27 of renal cell carcinoma^20^,^21^,^22^,^23^,^24^,^25^,^26^,^27^,^28^,^29^,^30^,^31^,^32^,^33^,^34^,^35^,^36^,^37^,^38^,^39^,^40^,^41^,^42^,^43^,^44^,^45^,^46^ and 11 of urothelial carcinoma originating from bladder (n = 6) and upper urinary tract (n = 5)^47^,^48^,^49^,^50^,^51^,^52^,^53^,^54^,^55^,^56^,^57^. The number of patients ranged from 30 to 1161 among the studies. As for the survival outcomes, 21 articles evaluated the association of CRP and OS. Prognostic role of CRP in CSS or disease specific survival (DSS) was evaluted in 25 articles.

### Outcome

**Figure 2:**
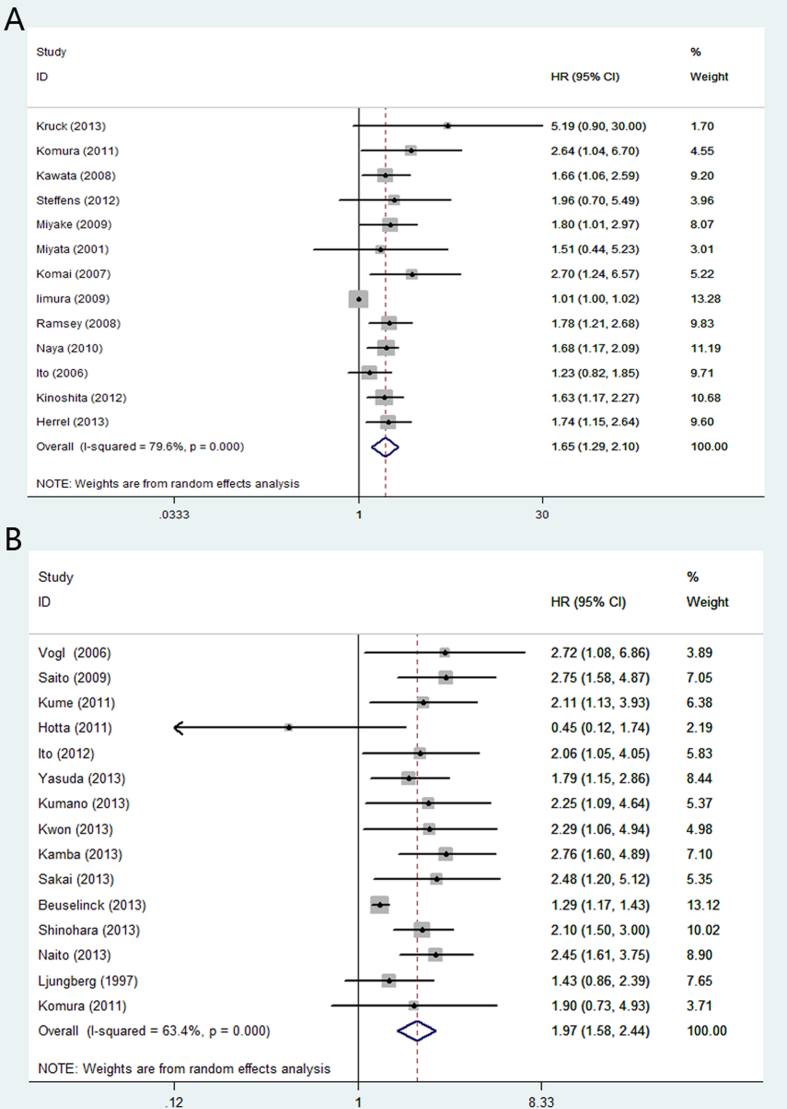
Meta-analysis of the association of CRP and clinical outcomes of patients with renal cell carcinoma. (**A**): CRP and CSS; (**B**): CRP and OS.

**Figure 3:**
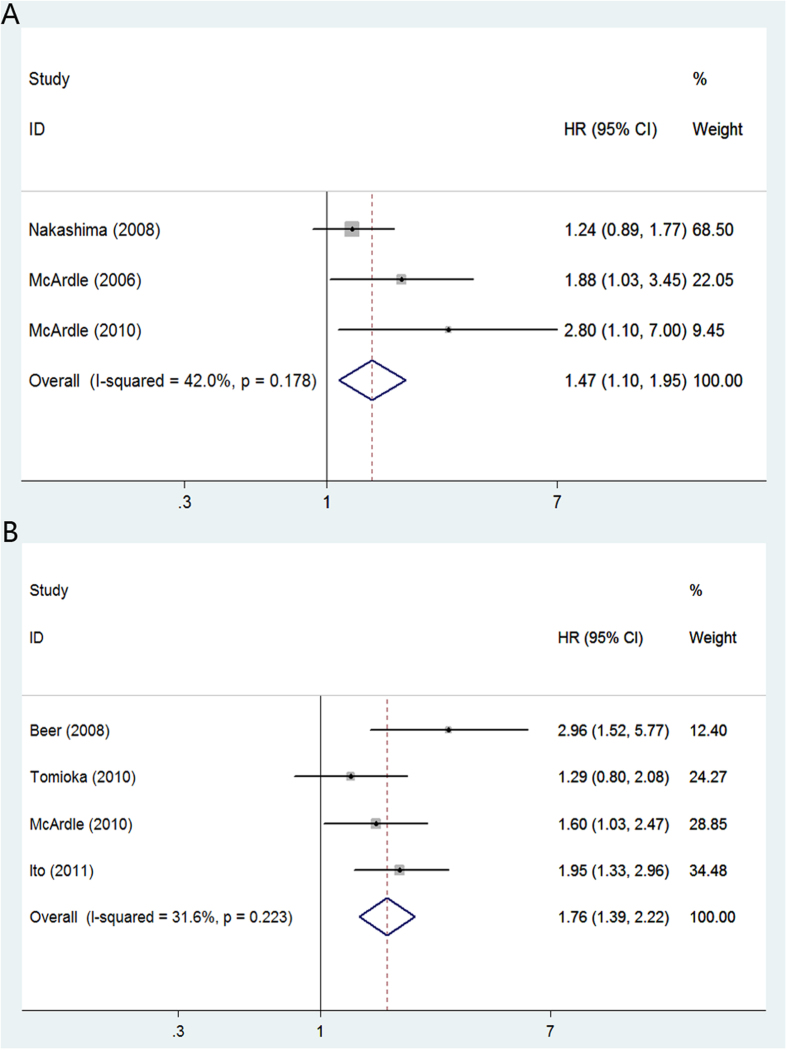
Meta-analysis of the association of CRP and clinical outcomes of patients with prostate cancer. (**A**): CRP and CSS; (**B**): CRP and OS.

**Figure 4:**
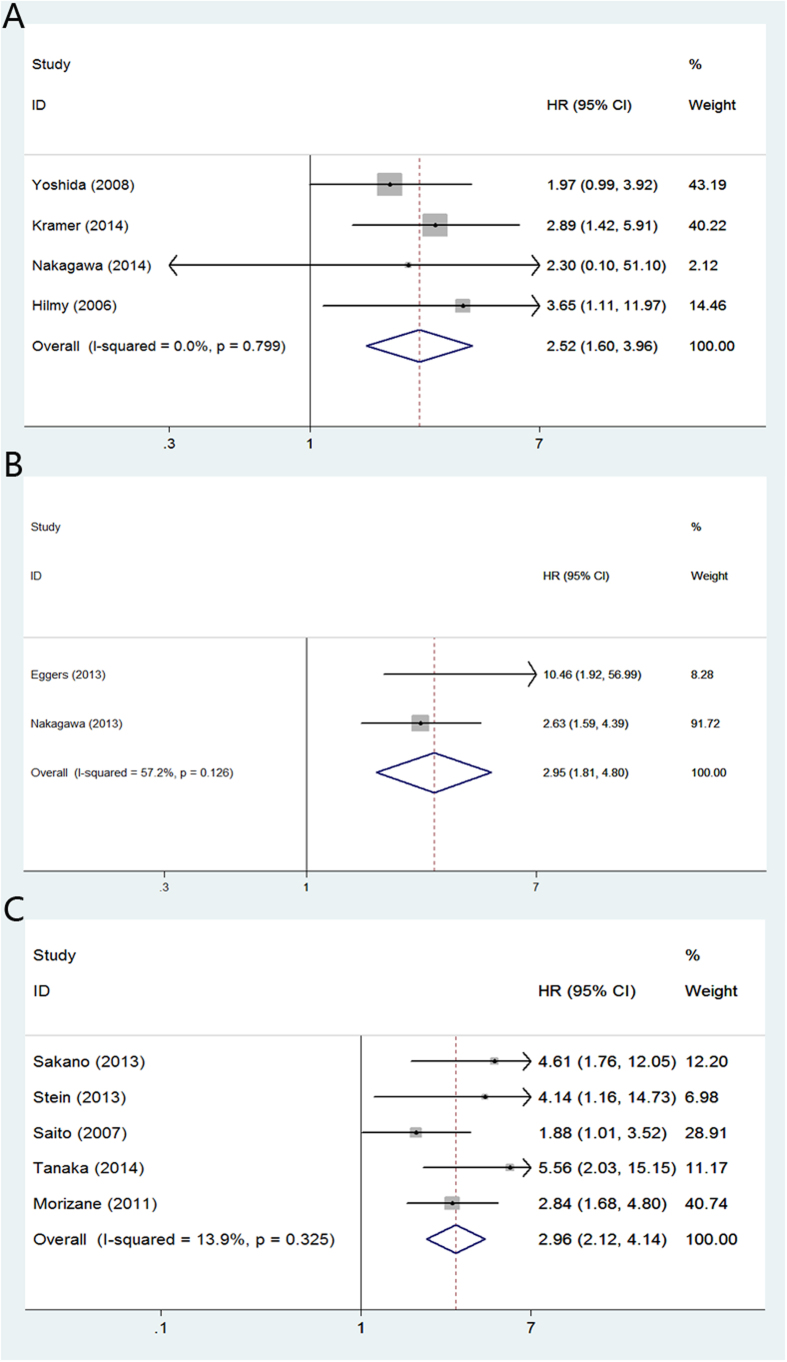
Meta-analysis of the association of CRP and clinical outcomes of patients with urothelial carcinoma or bladder cancer. (**A**): CRP and CSS in patients with bladder cancer; (**B**) CRP and OS in patients with bladder cancer; (**C**) CRP and CSS in patients with upper urinary tract urothelial carcinoma.

In renal cell carcinoma, high CRP yielded a worse CSS (random-effect model; [HR] = 1.65, 95% [CI] = 1.29–2.10; p < 0.01) (Fig. 2A) and OS (random-effects model; [HR] = 1.97, 95% [CI] = 1.58–2.44; p < 0.01) (Fig. 2B). The multivariable HRs (95% CI) of prostate cancer for CSS and OS were 1.91 (fixed-effect model; [HR] = 1.47; 95% CI = 1.10–1.95; p < 0.01) (Fig. 3A) and 1.77 (fixed-effects model; [HR] = 1.76, 95%CI = 1.39–2.22; p < 0.01) (Fig. 3B), respectively. For bladder cancer, the multivariable HRs (95% CI) for CSS and OS were 2.52 (fixed-effects model; [HR] = 2.52, 95% CI = 1.60–3.96; p < 0.01) (Fig. 4A) and 2.95 (fixed-effects model; [HR] = 2.95, 95% CI = 1.81–4.80; p = 0.03) (Fig. 4B). Five studies reported the prognostic role of CRP in upper urinary tract urothelial carcinoma and the multivariable HR for CSS was 2.96 (fixed-effects model; [HR] = 2.96, 95% CI = 2.12–4.14; p < 0.01) (Fig. 4C).

In order to explore whether CRP predicts the outcome of specific patients, we extracted the data on treatment and tumor stage. Among the 44 studies, only five studies including 763 localized RCC treated with nephrectomy evaluated the association between pretreatment CRP and CSS^22^,^24^,^30^,^38^,^58^. The multivariable HR (95% CI) for CSS was 3.66 (fixed-effect model; [HR] = 3.66, 95% CI = 2.20–6.09; p < 0.01) (Supplementary Figure S1A online). Another five studies included 585 cases and summarized that elevated pretreatment CRP was a poor predictor for OS in patients with metastatic RCC treated with molecular-targeted therapy^37^,^39^,^41^,^42^,^46^ (random-effects model; [HR] = 1.86, 95% CI = 1.30–2.66; p < 0.01) (Supplementary Figure S1B online). In addition, CRP also predicted a poor survival in metastatic prostate cancer no matter treated with chemotherapy^15^,^19^ (OS: fixed-effect model; [HR] = 2.18, 95% CI = 1.55–3.07; p < 0.01) (Supplementary Figure S1C online)or endocrine therapy^14^,^16^ (CSS: fixed-effect model; [HR] = 1.92, 95% CI = 1.22–3.03; p < 0.01) (Supplementary Figure S1D online). We identified two studies that reported the effect of CRP on CSS in patients with localized upper urinary tract urothelial carcinoma (UUT-UC) treated with nephroureterectomy and their combined HR was 1.46 (fixed-effect model; [HR] = 1.45, 95% CI = 1.12–1.88; p < 0.01) (Supplementary Figure S1E online)^48^,^53^.

### Subgroup analysis

As Table 2 showed, the meta-analysis of all studies suggested a significant association between baseline serum CRP and CSS (random-effects model; [HR] = 1.97, 95% [CI] = 1.59–2.45; p < 0.01). For overall survival, the multivariable HR (95% CI) of all studies was 1.99 [random-effects model; [HR] = 1.99, 95% [CI] = 1.66–2.38; p < 0.01).

Subgroup analyses by race showed that high CRP predicted a worse CSS (Caucasian: random-effects model; [HR] = 1.92, 95% CI = 1.34–2.75; p < 0.01; Asian: random -effects model; [HR] = 1.87, 95% CI = 1.55–2.26; p < 0.01). Similar role was shown for OS (Caucasian: random-effects model; [HR] = 1.74, 95% CI = 1.20–2.51; p < 0.01; Asian: fixed-effect model; [HR] = 2.10, 95% CI = 1.83–2.41; p < 0.01).

Studies were divided into two groups according to cutoff value. High CRP was shown to be a worse prognostic marker for CSS (cutoff ≤ 0.5: random-effects model; [HR] = 1.65, 95% CI = 1.33–2.04; p < 0.01; cutoff > 0.5: fixed-effect model; [HR] = 2.80, 95% CI = 2.14–3.66; p < 0.01) and OS (cutoff ≤ 0.5: random-effects model; [HR] = 1.88, 95% CI = 1.47–2.42; p < 0.01; cutoff > 0.5: fixed-effect model; [HR] = 2.06, 95% CI = 1.70–2.49; p < 0.01).

### Meta-regression analysis

We performed a meta-regression analysis to see the association between different cut-off values and HR (Supplementary Figure S2 online). For CSS, the results indicated that the association is greater when the cut-off value was higher (coefficient 0.68; p = 0.01). But no similar trend was observed in OS (coefficient 0.07; p = 0.56), which confirmed the results of subgroup analyses. To investigate the source of heterogeneity among studies, we conducted univariate meta-regression analyses by using variables as year of publication, race, tumor type, cutoff value and sample size suggested that the major sources of significant heterogeneity in studies on CSS were year of publication (coefficient = 0.07, p = 0.05, adjusted R^2^ = 0.13), tumor type (coefficient = 0.34, p = 0.02, adjusted R^2^ = 0.27) and cutoff value (coefficient = 0.68, p = 0.01, adjusted R^2^ = 0.33). For OS, only race (coefficient = −0.31, p = 0.06, adjusted R^2^ = 0.65) contributed to the heterogeneity. Then, a multiple meta-regression was carried out by using the five variables and we found these variables together could only explain heterogeneity in part (CSS: adjusted R^2^ = 42.4%; OS: adjusted R^2^ = 4.74%).

### Publication bias

**Figure 5:**
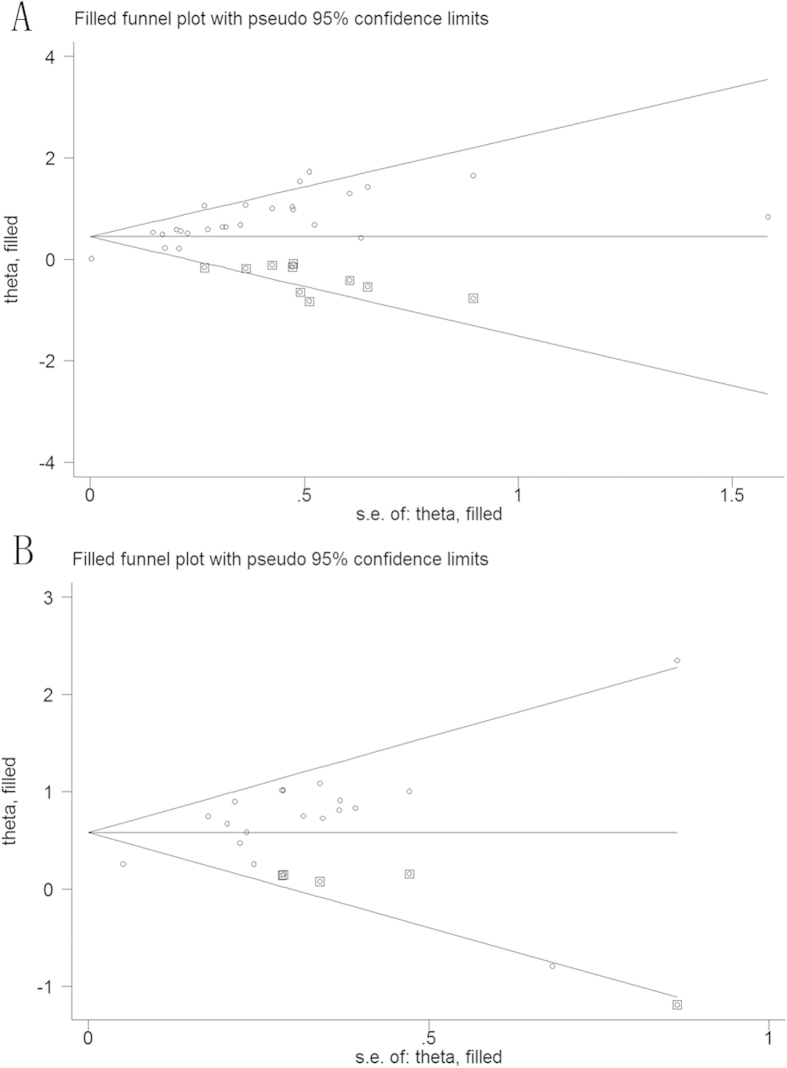
Funnel plot adjusted with trim and fill method for cancer specific survival (**A**) and overall survival (**B**). Circles: included studies. Diamonds: presumed missing studies.

Publication bias was evaluated using Begg’s funnel plot and the Egger’s linear regression test. However, significant publication bias was found for OS (p = 0.36 for Begg’s test and P < 0.01 for Egger’s test) and CSS (p = 0.09 for Begg’s test and p < 0.01 for Egger’s test). Then, we conducted funnel plots adjusted with trim and fill method. As shown in Fig. 5, ten theoretical studies were added in analysis of CSS and four in OS. The recalculated results did not change significantly for CSS (random-effects model; [HR] = 1.41, 95% CI = 1.26–1.58; p < 0.01) (Fig. 5A) and OS (random-effects model; [HR] = 1.49, 95% CI = 1.34–1.66; p < 0.01) (Fig. 5B), indicating the stability of the results.

## Discussion

To our knowledge, this is the most comprehensive meta-analysis studying the prognostic impact of CRP on urological cancers. Meanwhile, it is the first meta-analysis to evaluate the prognostic role of CRP in urothelial carcinoma/bladder cancer. Our results showed that elevated CRP was an independent prognostic marker in urological cancers, regardless of the tumor type, ethnicity background and cutoff value in the studies. Different pathological stages and treatment modalities could be correlated with the prognosis of cancers. However, few studies included in our meta-analysis analyzed these variables. In our meta-analysis, we evaluated the association between CRP and survival outcome of patients with different tumor stages or treated with different therapies. As we expected, the prognostic role of CRP was also confirmed in localized RCC treated with surgery, metastatic RCC treated with targeted therapy. Interestingly, the cutoff value of CRP should be carefully selected based on our results of meta-regression analysis. For CSS, the higher the cutoff value of CRP was, the higher HR was. However, there was no similar association between cutoff and overall survival in urological cancers.

The acute-phase reactant C-reactive protein is a member of the pentaxin protein family, mainly synthesized by hepatocytes, secreted into the circulation and mostly influenced by pro-inflammatory cytokines, particularly interleukin 6^59^. Apart from the liver, other tissues were able to synthesize the CRP. CRP is one of the major acute-phase proteins and is considered as a definitive marker of systemic inflammation. In clinical practice, it is commonly used to evaluate the severity of the systemic inflammation or outcomes of a variety of inflammation-related disorders^9^. In normal population, 70%–90% of samples have a CRP concentration of less than 0.3 mg/dl^60^, while serum CRP level of cancer patients is significantly higher^61^. A possible explanation is that inflammatory cytokines secreted by tumor cells could strongly stimulate the CRP production in liver^62^,^63^. Additionally, some tumor cells could also secret CRP and that may contribute to the serum CRP level^64^. Therefore, serum CRP level of cancer patients could be an indirect indicator of cancer related inflammation.

Previous studies reported that elevated CRP was associated with poor prognosis in several kinds of malignancies^6^,^8^. Since the links between cancer and inflammation were reported and accepted, clinical value of CRP in various malignancies was noticed by more and more researchers. However, the mechanism is not yet fully understood. As mentioned above, elevated CRP in serum represents a systemic inflammatory response, which may indicate much inflammatory mediators from cancer tissue. Meanwhile, inflammatory microenvironment in tumor could activate some specific signaling pathways critical for proliferation, angiogenesis and metastasis of cancer^65^. Therefore, a systematic inflammatory response defined by elevated serum CRP could be associated with prognosis of urological cancers.

Here, we wish to emphasize several limitations of our study. First, even though the amount of included studies is large, the heterogeneity is relatively high and could not be eliminated completely. The five variables included in our meta-regression analysis partly explained heterogeneity. Other factors, such as age and histological grade, is likely to affect the prognosis. Second, some studies of urothelial carcinoma contained both bladder cancer and urothelial carcinoma of upper urinary tract so that we have to combine the bladder cancer and urothelial carcinoma into one group. Third, owing to lack of data, the association between CRP and other clinical parameters such as pathological stage and prostate-specific antigen (PSA) was not explored. Above all, additional well-designed studies are necessary to present more reliable results in urological cancers.

## Conclusions

In conclusion, current evidence from the meta-analysis of published studies identified elevated baseline serum CRP as a strong prognostic biomarker in urological cancers. However, limitations listed above should be noted and more large-scale and standard investigations should be carried out.

## Methods

### Inclusion/exclusion criteria

Clinical studies were considered eligible if they investigated the association between pretreatment CRP in serum and survival outcome (OS, DSS or CSS) in patients who were diagnosed as urological cancer pathologically. No language or publication status restrictions were imposed. Studies were excluded based on any of the following criteria: (i) studies were review articles, laboratory articles or letters; (ii) HR was calculated with univariate rather than multivariable logistic regression analyses; (iii) papers lacked key information for calculation; (iv) a definite cutoff value of CRP wasn’t given; (v) if two studies were published by the same group with overlapping patient populations, the most recent one was selected.

### Literature search

Studies were identified by searching electronic databases. A systematic literature search was carried out using PubMed and Embase databases. The literature search was undertaken in May 2014, and updated in October 2014. No language restriction was applied. We used the following search terms to search related studies: CRP; C-reactive protein; C reactive protein; renal cell carcinoma; bladder cancer; prostate cancer; urothelial cancer; transitional cell carcinoma. Search strategies are as following: Pubmed: C-reactive protein [mesh] AND (urinary bladder neoplasms [mesh] OR prostatic neoplasms [mesh] OR kidney neoplasms [mesh] OR carcinoma, transitional Cell [mesh]); EMBASE: C-reactive protein AND (bladder cancer OR prostate cancer OR renal cell carcinoma OR urothelial cancer OR urothelial carcinoma OR transitional cell carcinoma).

### Study selection and quality assessment

After primary search, duplications are removed by screening the titles. Then, the titles and abstracts are reviewed for further evaluation. Finally, we will read the full-text for eligibility. If eligible, the study is included for the meta-analysis. Eligibility assessment was performed independently by two authors (L.Z. and X.C.). Disagreements were resolved by consensus. According to the review checklist of the Dutch Cochrane Centre proposed by Meta-analysis of Observational Studies in Epidemiology (MOOSE) group, we assessed the quality of all studies with the same method in Wu’s work^12^,^66^. The quality criteria include (1) clear definition of study population, (2) clear definition of study design, (3) enough sample size more than 30, (4) clear definition of outcome assessment, OS or CSS, (5) clear definition of cut-off of CRP level and (6) sufficient period of follow-up.

### Data extraction

Primary information, including hazard ratio (HR) and 95% confidence interval (CI) and p value, was extracted directly from articles by two investigators independently. Additional data were extracted from the studies, including the first author, year of publication, sample size, race, cutoff value and other clinical characteristics.

### Statistical analysis

The primary outcome measure was the hazard ratios of CRP predicting CSS, DSS or OS, which were obtained from each study. We combined CSS and DSS in an analysis due to similar definitions. Meta-analysis was performed to calculate the estimated hazard ratio and its variance. Results were shown in forest plot graphs. HR greater than 1 and 95% CI for the aggregated HR not crossing 1 indicates a prognostic role of elevated CRP. P < 0.05 was considered statistically significant and all P values were two-sided. Heterogeneity was defined as p < 0.10 or I^2 ^> 50%. Random effects analysis of the Mantel Haenszel Model was utilized when statistical heterogeneity was suggested (p < 0.10 or I^2 ^> 50%). If not, a fixed effect model was used. If statistical heterogeneity was found (p < 0.10 or I2 > 50%), subgroup analysis and meta-regression were conducted to explore the potential source of heterogeneity among studies. In our study, subgroup analyses and meta-regression were conducted for tumor, race and cutoff value. Egger’s test and Begg’s test were performed to test for publication bias. Statistical analyses were done with Stata version 12.0 (STATA Corporation, College Station, TX, USA).

## Additional Information

**How to cite this article**: Zhou, L. *et al.* Prognostic Role of C-Reactive Protein In Urological Cancers: A Meta-Analysis. *Sci. Rep.* **5**, 12733; doi: 10.1038/srep12733 (2015).

## Supplementary Material

Supplementary Information

## Figures and Tables

**Table 1:** Characteristics of all identified studies.

Author	Year	Country	Tumor	N	Male	Metastatic	Outcome	Cut-off (mg/dl)
Nakagawa	2014	Japan	BC	267	212	267	CSS	0.5
Kramer	2014	Germany	BC	194	151	25	CSS	0.5
Tanaka	2014	Japan	UUT-UC	564	409	122	CSS	0.5
Sakano	2013	Japan	UUT-UC	536	370	0	DSS	0.13
Kruck	2013	Germany	RCC	327	219	NA	CSS	0.25
Kamba	2013	Japan	RCC	144	112	144	OS	0.3
Shinohara	2013	Japan	RCC	473	329	473	OS	0.3
Nakagawa	2013	Japan	BC	114	92	95	OS	0.5
Beuselinck	2013	Belgium	RCC	200	142	200	OS	0.5
Kwon	2013	Korea	RCC	106	78	106	OS	0.5
Stein	2013	Germany	UUT-UC	115	83	26	CSS	0.5
Kumano	2013	Japan	RCC	83	61	83	OS	0.8
Yasuda	2013	Japan	RCC	52	42	52	OS	0.8
Naito	2013	Japan	RCC	556	411	556	OS	1
Herrel	2013	America	RCC	284	176	0	CSS	2
Sakai	2013	Japan	RCC	164	130	164	OS	4
Eggers	2013	Germany	BC	34	28	34	OS	0.8
Steffens	2012	Germany	RCC	1,161	761	226	CSS	0.4
Kinoshita	2012	Japan	RCC	559	NR	559	CSS	1
Ito	2012	Japan	RCC	181	133	181	OS	1.8
Komura	2011	Japan	RCC	170	114	0	CSS,OS	0.3
Kume	2011	Japan	RCC	94	NR	94	OS	0.3
Ito	2011	Japan	PC	80	80	80	OS	0.5
Hotta	2011	Japan	RCC	105	76	18	OS	0.5
Morizane	2011	Japan	UUT-UC	30	23	30	CSS	1
Tomioka	2010	Japan	PC	287	287	265	OS	0.3
Naya	2010	Japan	RCC	117	NR	0	CSS	1
McArdle	2010	UK	PC	98	98	0	CSS,OS	1
Iimura	2009	Japan	RCC	249	170	27	CSS	0.5
Miyake	2009	Japan	RCC	52	35	52	CSS	0.5
Saito	2009	Japan	RCC	108	80	108	OS	0.5
Nakashima	2008	Japan	PC	126	126	126	DSS	0.15
Kawata	2008	Japan	RCC	252	196	NA	CSS	0.3
Yoshida	2008	Japan	BC	88	63	NA	CSS	0.5
Beer	2008	Canada	PC	160	160	160	OS	0.8
Ramsey	2008	UK	RCC	83	55	NA	CSS	1
Komai	2007	Japan	RCC	101	63	0	DSS	0.5
Saito	2007	Japan	UUT-UC	130	88	NA	DSS	0.5
Vogl	2006	Japan	RCC	99	74	99	OS	0.8
Hilmy	2006	UK	BC	103	70	0	CSS	1
McArdle	2006	UK	PC	62	62	62	CSS	1
Ito	2006	Japan	RCC	178	38	0	CSS	1
Miyata	2001	Japan	RCC	92	71	19	CSS	0.5
Ljungberg	1997	Sweden	RCC	196	119	66	OS	1

N, number of patients. Metastatic, number of patients with metastatic cancer. Cut-off: cut-off value of c-reactive protein (CRP) applied in each study. BC, bladder cancer. RCC, renal cell carcinoma. UUT-UC, upper urinary tract - urothelial carcinoma. DSS, disease specific survival. CSS, cancer specific survival. OS, overall survival. NA, not available.

**Table 2:** Main results of subgroup analyses.

Outcome	Variable	Number of studies	Model	HR(95%,CI)	I^2^	P_heterogeneity_
CSS	All	25	Random	1.97(1.59,2.45)	81.5%	<0.01
	Race					
	Caucasian	9	Random	1.92(1.34,2.75)	82.7%	<0.01
	Asian	16	Random	1.87(1.55,2.26)	33.7%	<0.01
	Cut-off					
	≤0.5	16	Random	1.65(1.33,2.04)	78.2%	<0.01
	>0.5	9	Fixed	2.80(2.14,3.66)	0.0%	0.65
OS	All	21	Random	1.99(1.66,2.38)	62.6%	<0.01
	Race					
	Caucasian	5	Random	1.74(1.20,2.51)	67.5%	0.02
	Asian	16	Fixed	2.10(1.83,2.41)	0.0%	0.57
	Cut-off					
	≤0.5	11	Random	1.88(1.47,2.42)	69.9%	<0.01
	>0.5	10	Fixed	2.06(1.70,2.49)	5.7%	0.39

Random: random-effects model. Fixed: fixed-effects model. P_heterogeneity_, P value for Cochran’s Q test of heterogeneity.
