# Supplementary material for: Prognostic Role of C-Reactive Protein In Urological Cancers: A Meta-Analysis
Source: Sci Rep. 2015 Aug 3;5:12733. doi: 10.1038/srep12733 (PMC4522672; doi:10.1038/srep12733)

Title: Prognostic Role of C-Reactive Protein In Urological Cancers: A Meta-Analysis

Authors: Liang Zhou, Xiang Cai, Qiang Liu, Zhong-Yu Jian, Hong Li and Kun-Jie Wang*

Figure legends:

Supplementary Figure S1. Meta-analyses of the association of CRP and clinical outcomes of specific patient groups with different treatments.
(A) CRP and CSS in localized RCC treated with nephrectomy
(B) CRP and OS in metastatic RCC treated with molecular-targeted therapy
(C) CRP and CSS in metastatic prostate cancer treated with endocrine therapy
(D) CRP and OS in metastatic prostate cancer treated with chemotherapy.
(E) CRP and CSS in localized urothelial carcinoma with nephroureterectomy;
CSS: cancer specific survival; OS: overall survival; RCC: renal cell carcinoma; CRP: c-reactive protein.

Supplementary Figure S2. Association between lnHR (hazard ratio) and cutoff value of CRP in all included studies. A: Meta-regression analysis between lnHR for cancer specific survival and cutoff value (coefficient 0.68; p = 0.01); B: Meta-regression analysis between lnHR for overall survival and cutoff value (coefficient 0.07; p = 0.56).

Supplementary Figure S1

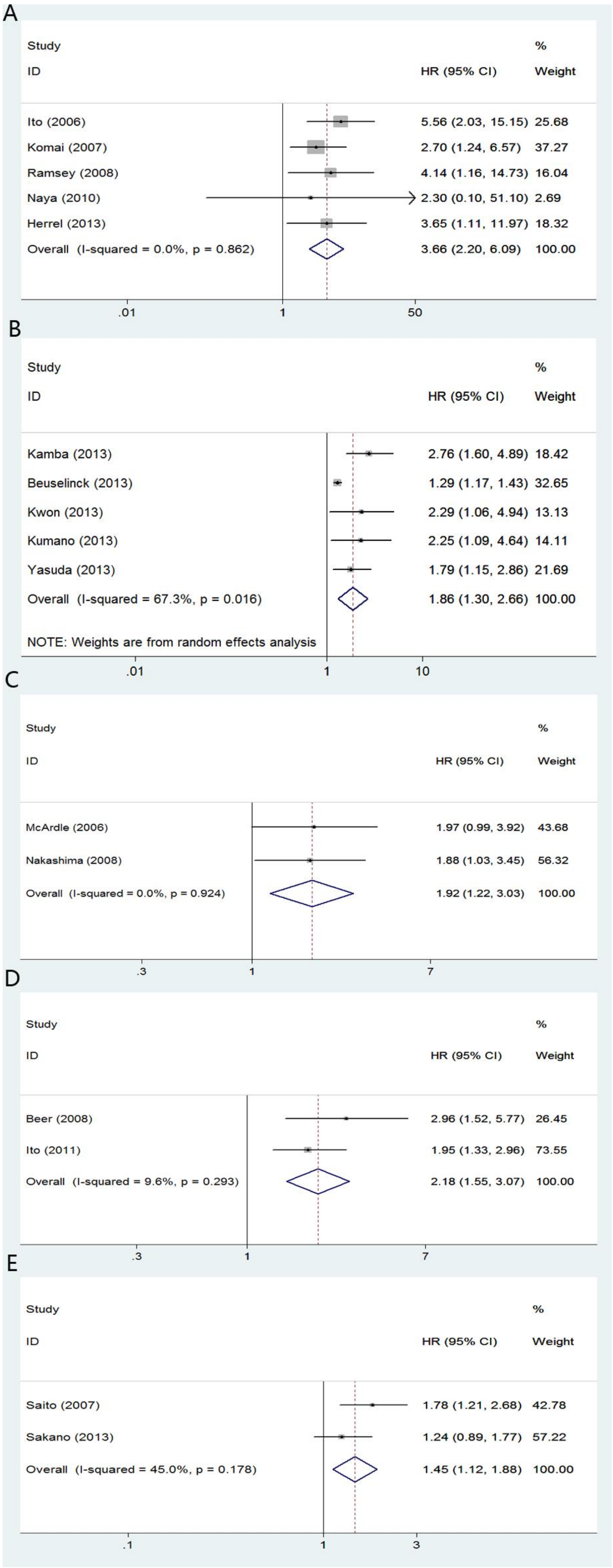

Supplementary Figure S2

A

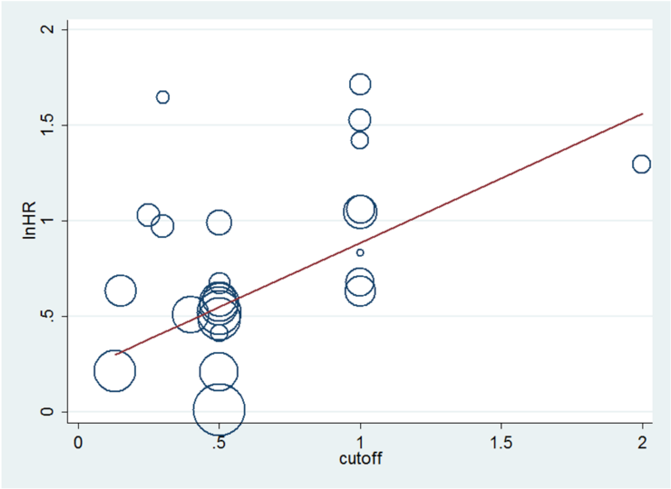

B

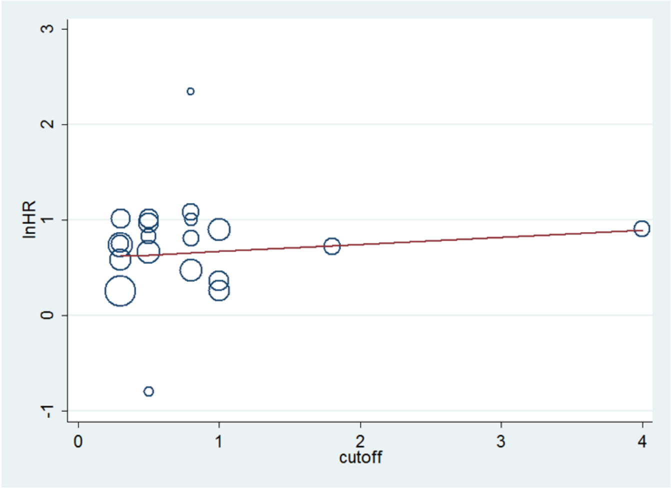

Supplement: Supplementary Information [file srep12733-s1.pdf]
